# Cortical Spectral Activity and Connectivity during Active and Viewed Arm and Leg Movement

**DOI:** 10.3389/fnins.2016.00091

**Published:** 2016-03-10

**Authors:** Julia E. Kline, Helen J. Huang, Kristine L. Snyder, Daniel P. Ferris

**Affiliations:** ^1^Department of Biomedical Engineering, University of MichiganAnn Arbor, MI, USA; ^2^School of Kinesiology, University of MichiganAnn Arbor, MI, USA

**Keywords:** EEG, ICA, motor control, viewed movement, connectivity

## Abstract

Active and viewed limb movement activate many similar neural pathways, however, to date most comparison studies have focused on subjects making small, discrete movements of the hands and feet. The purpose of this study was to determine if high-density electroencephalography (EEG) could detect differences in cortical activity and connectivity during active and viewed rhythmic arm and leg movements in humans. Our primary hypothesis was that we would detect similar but weaker electrocortical spectral fluctuations and effective connectivity fluctuations during viewed limb exercise compared to active limb exercise due to the similarities in neural recruitment. A secondary hypothesis was that we would record stronger cortical spectral fluctuations for arm exercise compared to leg exercise, because rhythmic arm exercise would be more dependent on supraspinal control than rhythmic leg exercise. We recorded EEG data while ten young healthy subjects exercised on a recumbent stepper with: (1) both arms and legs, (2) just legs, and (3) just arms. Subjects also viewed video playback of themselves or another individual performing the same exercises. We performed independent component analysis, dipole fitting, spectral analysis, and effective connectivity analysis on the data. Cortical areas comprising the premotor and supplementary motor cortex, the anterior cingulate, the posterior cingulate, and the parietal cortex exhibited significant spectral fluctuations during rhythmic limb exercise. These fluctuations tended to be greater for the arms exercise conditions than for the legs only exercise condition, which suggests that human rhythmic arm movements are under stronger cortical control than rhythmic leg movements. We did not find consistent spectral fluctuations in these areas during the viewed conditions, but effective connectivity fluctuated at harmonics of the exercise frequency during both active and viewed rhythmic limb exercise. The right premotor and supplementary motor cortex drove the network. These results suggest that a similarly interconnected neural network is in operation during active and viewed human rhythmic limb movement.

## Introduction

An interesting feature of human neurophysiology is that active movement and viewed movement recruit many of the same neural structures (Prinz, 1997). “Mirror neurons,” which fire in response to both active and viewed movements were first discovered in monkeys (Di Pellegrino et al., 1992). Since then, a slew of neuroimaging studies have confirmed that a similar neural mechanism exists in humans. Electroencephalography (EEG) studies have demonstrated desynchronization in the human motor cortex during both active and viewed movement (Cochin et al., 1998, 1999; Calmels et al., 2006; Avanzini et al., 2012; Cevallos et al., 2015). Studies with functional magnetic resonance imaging (fMRI) have found overlapping cortical activation during active and viewed movement (Iacoboni et al., 1999; Buccino et al., 2001; Grezes et al., 2003; Manthey et al., 2003).

Humans regularly coordinate arm and leg movements during locomotion or locomotion-like movements. Despite the substantial literature on viewed motion, most studies comparing neural activity during active and viewed movements focus on isolated hand or foot movements, rather than full-body rhythmic limb movement. Human full-body rhythmic limb movement likely involves a distribution of cortical and spinal control, which may make viewed rhythmic arm and leg movements substantially different from viewed hand or foot movements. In quadrupedal animals, the coordination of rhythmic limb behaviors relies heavily on collections of oscillatory neurons in the spinal cord, known as central pattern generators (Brown, 1914; Grillner, 1975; Grillner et al., 1995; Marder and Calabrese, 1996; Duysens and van de Crommert, 1998; Juvin et al., 2005; Rossignol et al., 2006). In humans, the evidence suggests that rhythmic limb movements are under both spinal and cortical control. Functional near infrared spectroscopy studies (Miyai et al., 2001; Harada et al., 2009), transcranial magnetic stimulation studies (Petersen et al., 2001, 2003), and electroencephalography (EEG) studies (Gwin et al., 2011; Severens et al., 2012; Sipp et al., 2013) have shown cortical activation during human steady-state walking. There is also indirect evidence for central pattern generators in humans. This evidence includes primitive stepping-like motions in infants (Yang et al., 2005), rhythmic lower limb contractions in a patient with a complete spinal cord injury when the limbs are moved through the motion of gait (Wernig and Phys, 1992; Dobkin et al., 1995), and vibration-induced air stepping in healthy subjects (Isaev et al., 2004). Because humans likely share features of quadrupedal neural control but have adapted to become predominantly bipedal, there may be differences in the relative contributions of cortical and spinal control during rhythmic movement involving the arms and legs.

One way to examine cortical control is to use effective connectivity to find the causal relationship between brain regions. Positron emission tomography (Cabeza et al., 1997; Rosenbaum et al., 2010) and fMRI (Biswal et al., 1995; Greicius et al., 2003; Kiran et al., 2015) are frequently used to study effective connectivity. However, these modalities require participants to remain stationary and therefore cannot examine brain connectivity during unconstrained full-body motion. Another approach for studying real-world activities is to use high-density EEG, independent component analysis (ICA), and source localization techniques (Gwin et al., 2010, 2011; Gramann et al., 2011; Sipp et al., 2013; Kline et al., 2014). Although this EEG approach does not have the spatial resolution of fMRI connectivity studies, it does provide excellent temporal resolution with spatial resolution of around a few centimeters (Mullen et al., 2011). Lau and colleagues combined high-density EEG, ICA, and source localization with Granger causality to show that sensorimotor cortical connectivity was greater for standing compared to walking (Lau et al., 2012). Using a similar approach may provide additional insight into brain function as it relates to active and viewed movement.

The purpose of this study was to quantify the differences in cortical spectral fluctuations and effective connectivity during active and viewed full-body rhythmic limb movements. Our overall hypothesis was that we would be able to detect similar but weaker electrocortical spectral fluctuations and effective connectivity during viewed limb movement compared to active limb movement due to the similarities in neural recruitment. We tested different combinations of arm and leg movements (arms and legs, legs only, and arms only), because we hypothesized that active rhythmic leg-only movements would show little spectral fluctuations based on evidence that suggests that rhythmic leg movements likely use more spinal control (Sakamoto et al., 2007, 2014). Additionally, some have suggested that the mirror neuron system is highly involved in human social interaction (Gallese et al., 2004; Oberman et al., 2007). Therefore, humans may have differences in cortical activity when viewing themselves compared to viewing someone else perform a movement. If there are indeed differences in electrocortical spectral fluctuations for different combinations of active arms and legs movements or viewing perspective, we hypothesized that there would also be similar relative differences in effective connectivity for viewing those arm and leg movements. To test these hypotheses, we had subjects perform different combinations of arm and leg rhythmic movements on a recumbent stepper while we videotaped them. The subjects later viewed video playback of themselves and another individual performing the movements. We recorded scalp EEG data for all conditions.

## Materials and methods

### Subjects and experimental setup

Ten healthy adults (mean age 25.6 ± 4.4, 5 females) with no history of neurological disease or musculoskeletal injuries participated in this study. All participants signed a consent form approved by the University of Michigan Institutional Review Board.

We used a customized recumbent stepping machine (TRS 4000, NuStep, Ann Arbor, MI) with an adjustable level of resistance and an isokinetic motor (Huang and Ferris, 2009). The stepping machine combined features of a stair stepper and a recumbent bicycle. The handles and pedals were coupled so that the left handle and right pedal move together.

Before data collection, we fitted the subjects with a 256-channel EEG cap (Biosemi ActiveTwo, Amsterdam, Netherlands). We digitized the position of each electrode relative to the subject's head using a digitizer (Zebris, Germany). All electrode offsets were < 20 mV. We recorded EEG data at 512 Hz for all conditions.

**Figure 1:**
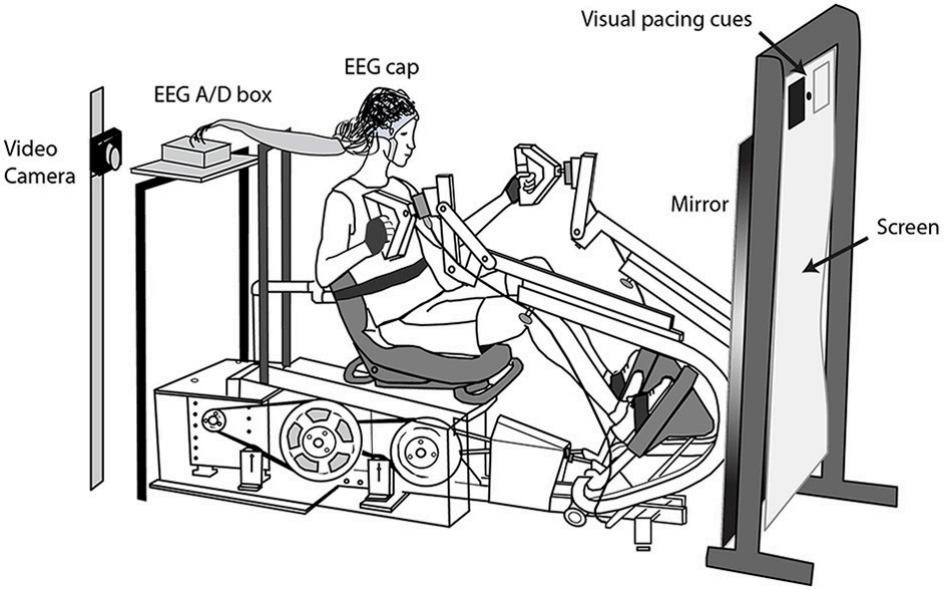
**Experimental setup**. Subjects moved to the pace of visual cues with (1) both their arms and legs, (2) their legs only, and (3) their arms only. A video camera to the left on the subject's head recorded videos of the subject exercising, as viewed in the mirror, for the three active conditions. For the viewed conditions, we removed the mirror, and projected life-size video playbacks of the subject or another individual exercising. The subjects remained seated in the stepping device during the viewed conditions. We recorded EEG during all conditions.

Subjects initially practiced stepping on the device at a range of resistances. We let the subjects choose the resistance at a level that they deemed challenging but not uncomfortable. We secured a Velcro strap around the subject's midsection to minimize torso movement and strapped the subject's feet to the pedals (they remained strapped to the pedals for the two active leg conditions). We placed the EEG amplifier on a platform directly behind the subject and draped the electrode leads over a bar (Figure 1). Directly in front of the subject, we placed a large mirror (79 × 168 cm). A screen above the mirror displayed visual cues that set the pace of the movement. We mounted a video camera at the subject's eye level, ~6 inches to the left of the head, pointed at the mirror. This allowed us to record videos of the subject exercising from the subject's viewpoint. We used a mirror in front of the subjects so that the view for all conditions was similar and followed a common practice in gait rehabilitation (Behrman and Harkema, 2000).

### Data collection

The subjects performed rhythmic arm and leg movement on the device (active conditions) and then sat quietly and viewed video playbacks of themselves and another individual performing the same movements (viewed conditions). During the active conditions, we also recorded position data based on the motor position signal of the recumbent stepper. The maximum motor position signal corresponded to the right pedal being fully extended. We synchronized the data streams using a square wave of constant frequency sent simultaneously to all recording systems.

The subjects moved on the device in three different ways in the following order: (1) with both their arms and their legs, (2) with their legs only, and (3) with their arms only. During the legs only condition, the subjects moved with their hands folded comfortably in their lap. During the arms only condition, the subjects moved with their feet resting on the floor, or on foot rests for shorter subjects.

The visual cues paced the subjects to move at 70 arm or leg extensions per minute. The cues consisted of a pair of squares at opposite sides of a central fixation point. The squares shaded from white to black at a fixed rate (1.16 Hz) and were 180° out of phase with each other. The subjects kept their eyes on the fixation point and moved so that the corresponding limb was fully-extended when the left or right square turned black. For the combined arms and legs condition, legs were given precedent, and the subjects moved so that the corresponding leg was fully extended when a square turned black. The subjects were allowed to practice exercising in synchrony with the cues as needed. We recorded a 5-min video of the subject exercising, as seen in the mirror, during each active condition.

During the viewed conditions, we removed the mirror that had been in front of a video projection screen (84 × 165 cm; Figure 1). On the screen, we then played a total of seven 5-min videos to the subject in a randomized order. The videos were: (1) playback of the subject exercising with both their arms and legs, (2) playback of the subject exercising with their legs only, (3) playback of the subject exercising with their arms only, (4–6) pre-recorded videos of another individual exercising in the three ways described above, (7) a control video of the recumbent stepper moving on its own, with no one seated in it. The video playback was adjusted to be life-size. During the viewed conditions, we gave the subjects a fixation point on the center of the screen, roughly at the center of the torso of the individual in the video, to prevent large eye movements during viewing.

### Data processing

We post-processed the EEG signals using custom scripts in EEGLAB (Delorme and Makeig, 2004). First, we merged the EEG recordings into a single dataset and high-pass filtered above 1 Hz to remove drift. We rejected channels exhibiting substantial artifact based on the methods of Gwin et al. (2010). These rejection guidelines did not reject enough channels to ensure good convergence of our ICA algorithm, therefore we altered the cutoffs slightly to reject an average of 140 channels per subject and re-referenced the remaining channels to a common average reference. Next, we rejected EEG time windows with high artifact across all channels based on visual inspection. To these cleaned datasets we applied infomax (Bell and Sejnowski, 1995) independent component analysis (ICA) as implemented on GPU by CUDAICA (Raimondo et al., 2012). This parsed the data into spatially fixed, temporally independent component (IC) signals (Makeig et al., 1996). The EEGLAB DIPFIT function (Oostenveld and Oostendorp, 2002) modeled each IC as an equivalent current dipole within a boundary element head model based on the MNI brain (Montreal Neurological Institute, MNI, Quebec). ICs with a best-fit equivalent current dipole that accounted for < 85% of the variance seen at the scalp were excluded from further analysis (Gwin et al., 2011).

We clustered the remaining ICs from all 10 subjects using a k-means clustering algorithm on vectors describing similarities in dipole location, scalp topography, and spectra (Gwin et al., 2011). If clusters contained ICs from five or fewer subjects or if their location and/or average scalp map were indicative of eye movement or muscle activity (Jung et al., 2000a,b), we excluded them from further analysis. For each electrocortical cluster and condition, we created an event-locked plot of spectral power fluctuation (Makeig, 1993; Gwin et al., 2011). Our data epochs began at full extension for one arm or leg (active or viewed) and ended at subsequent full extension for the same arm or leg. For each epoch, we computed single trial spectrograms. To ensure that each extension event occurred at the same latency in every trial, we linearly warped each single trial spectrogram. We averaged these spectrograms over trials for each IC and over ICs for each cluster. For each cluster and condition, we subtracted the average log spectrum across all time points from the log spectrum for each individual time point, to easily visualize spectral changes from baseline. These plots of spectral fluctuation are called event-related spectral perturbation (ERSP) plots. Bootstrapping methods available in EEGLAB (Delorme and Makeig, 2004) determined regions of significant difference from baseline for the ERSP plots (*p* < 0.05).

To quantify the spectral differences between the active conditions, we computed grand mean log power spectra for each exercise condition (arms only, arms and legs, legs only) for each independent component cluster. For each cluster, we used Wilcoxon rank sum tests in MATLAB for each frequency band of interest (frequency resolution = 0.026 Hz) to evaluate significant mean power differences between pairs of conditions (*p* < 0.05).

### Connectivity analysis

We also performed effective connectivity analysis on the epochs of data described above. Using the EEGLAB-compatible SIFT toolbox (Delorme et al., 2011), we created a custom data analysis pipeline. The preprocessing pipeline involved first downsampling the data to 128 Hz and piecewise linearly detrending using a 330 ms window every 82.5 ms. Next, we used the Hannan-Quinn, Swartz Baysian, and Akaike Information Criteria to determine the appropriate model order within a 200 ms sliding windows every 54.7 ms. A Vieira-Morf lattice algorithm available in SIFT fit the multivariate autoregressive (MVAR) model. Examining the eigen-values of the MVAR coefficient matrix allowed us to determine if the model was stable. We checked the whiteness of the model by multiple measures including the Ljung-Box test, the Box-Pierce test, the McLeod-Li test, and the Autocorrelation Function (ACF) test. The smallest model order that lead to stability and whiteness was the desired outcome. A model order between 1 and 3 satisfied these criteria for our data for all subjects and all conditions. With these MVAR models, we calculated connectivity and connectivity direction using directed transfer function (Kaminski and Blinowska, 1991). Directed transfer function is generally robust to both noise and indirect connections. To test the significance of the connectivity fluctuations, we used bootstrap significance testing with 200 resamples.

Furthermore, we wished to determine which cluster pairs and conditions had the greatest effective connectivity. We found the maximum connectivity value for each cluster pair at each condition. We determined which cluster pair/condition combinations had maximum connectivity values at least a standard deviation greater than the mean for all cluster pair/condition combinations. These cluster pair/condition combinations are referred to as having “supratheshold connectivity.”

We also wanted to quantify the rate at which the average connectivity in the cortical network changed over time. For each condition, we took fast Fourier transform of the connectivity values across time for each frequency. Because our measure of interest was the relative power at different frequencies, we used a zero-padded window of 128 samples, and took the magnitude of the FFT-value to determine the power spectrum for each frequency value from 0 to 18 Hz. We then took the mean of the resultant power spectrum over frequencies and component pairs to get an overall measure of the frequencies at which connectivity fluctuated for each condition.

## Results

### EEG results

**Figure 2:**
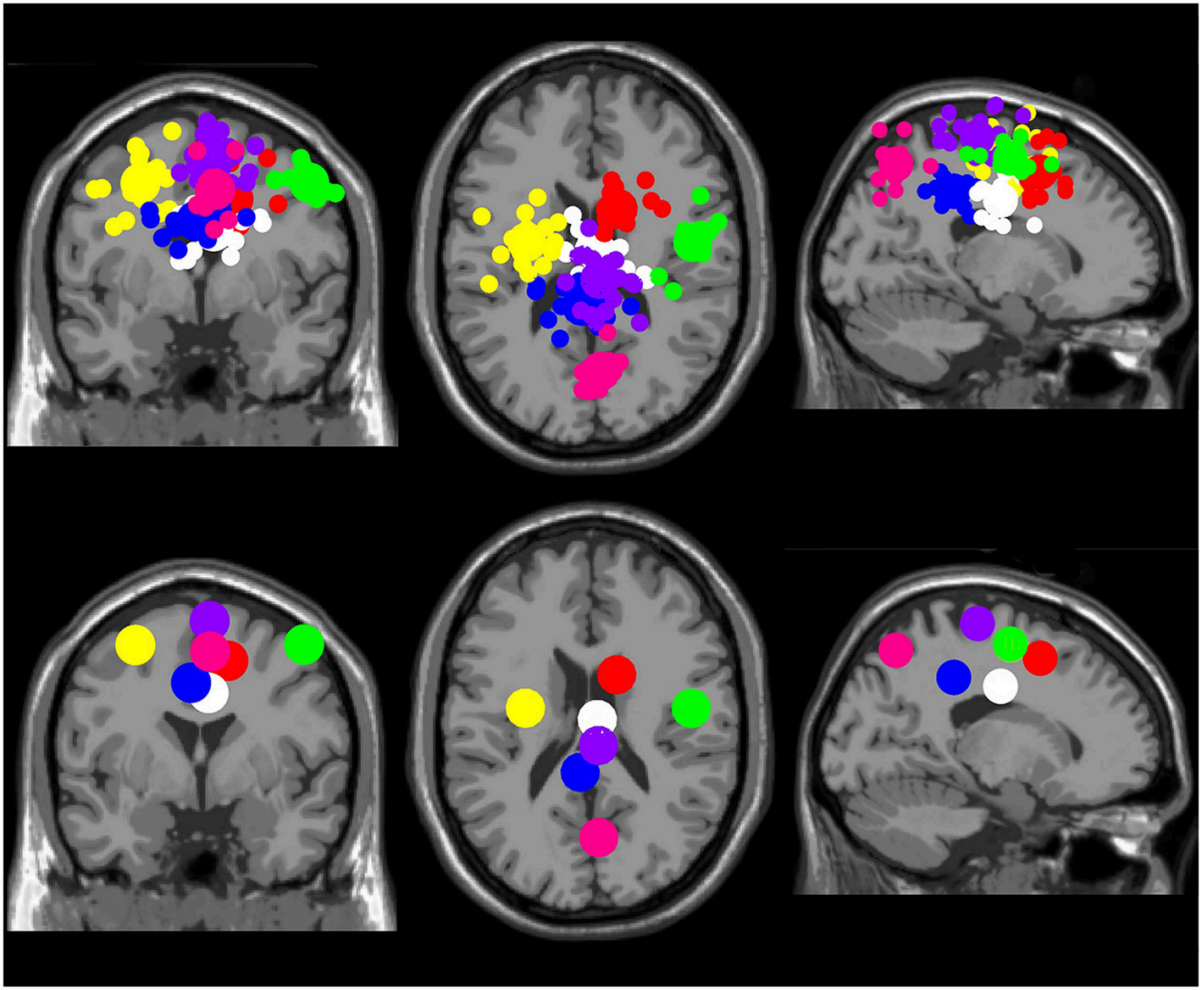
**Electrocortical clusters**. Clusters containing electrocortical sources from at least 5 of 10 subjects. Yellow is left premotor and supplementary motor cortex, purple is middle premotor, and supplementary motor cortex, green is right premotor and supplementary motor cortex, red is right anterior cingulate, white is middle anterior cingulate, blue is middle posterior cingulate, and pink is middle parietal cortex. From left to right, the top three images show the independent component dipoles for each cluster from a coronal, horizontal, and sagittal perspective, respectively. The bottom three images show the centroid locations for each cluster from the same three perspectives.

**Figure 3:**
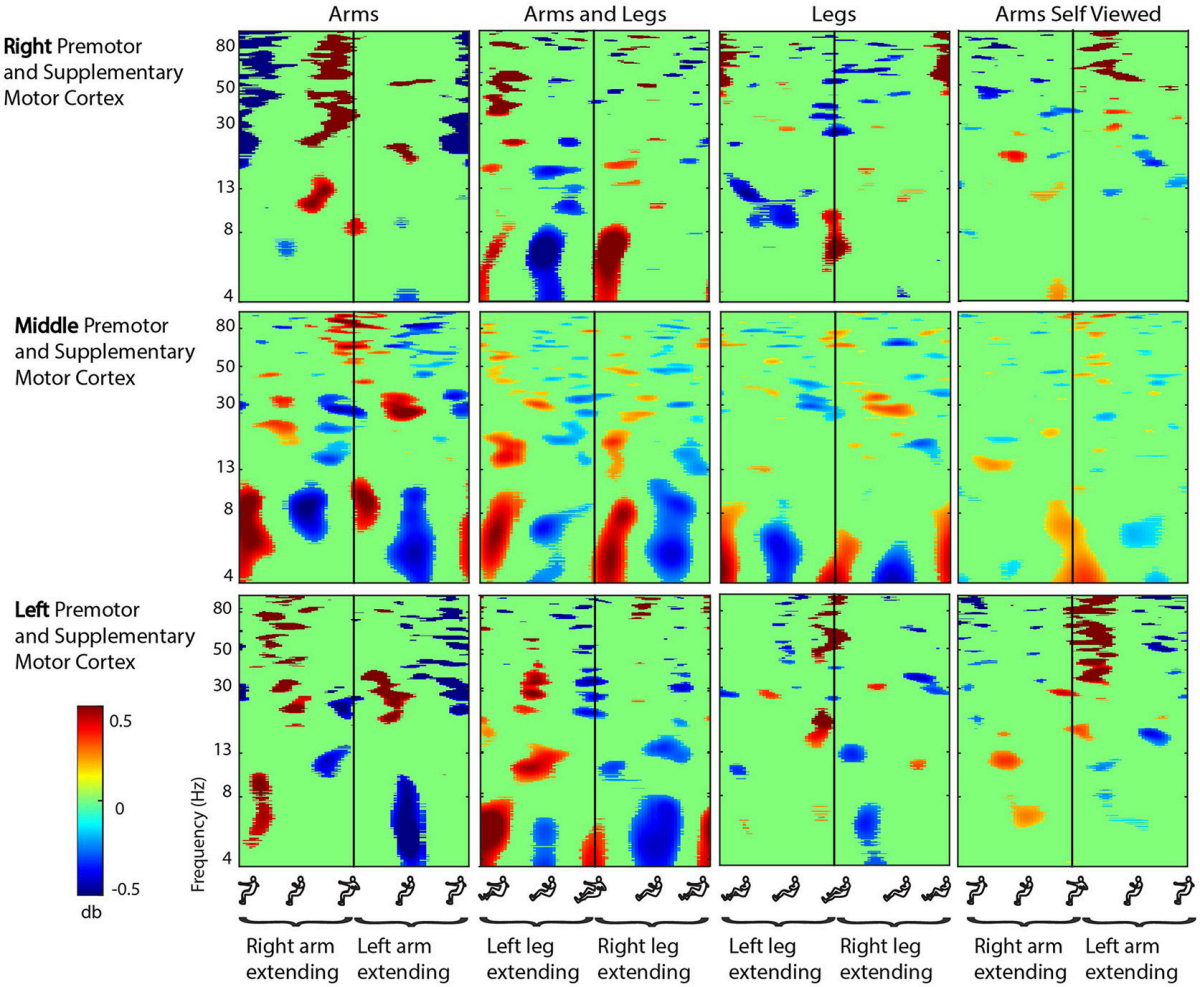
**Motor cortex ERSPs**. Event-related spectral perturbation (ERSP) plots showing change in spectral power during rhythmic arm and leg movement in the right, middle, and left premotor and supplementary motor cortex. From left to right, subjects moved with their arms only, both their arms and legs, their legs only, and viewed video playback of themselves moving with just their arms. Each row represents a cortical area, and each column represents a condition. For all plots, red represents a power increase from baseline, and blue represents a power decrease from baseline. We set non-significant differences to 0 dB (green). All ERSPs start and end with the same limb fully extended. The figure outlines on the x axis indicate the phase of movement, and the written labels indicate when each limb was extending. Note: for the arms and legs condition, the left leg and right arm extended together, and vice versa.

**Figure 4:**
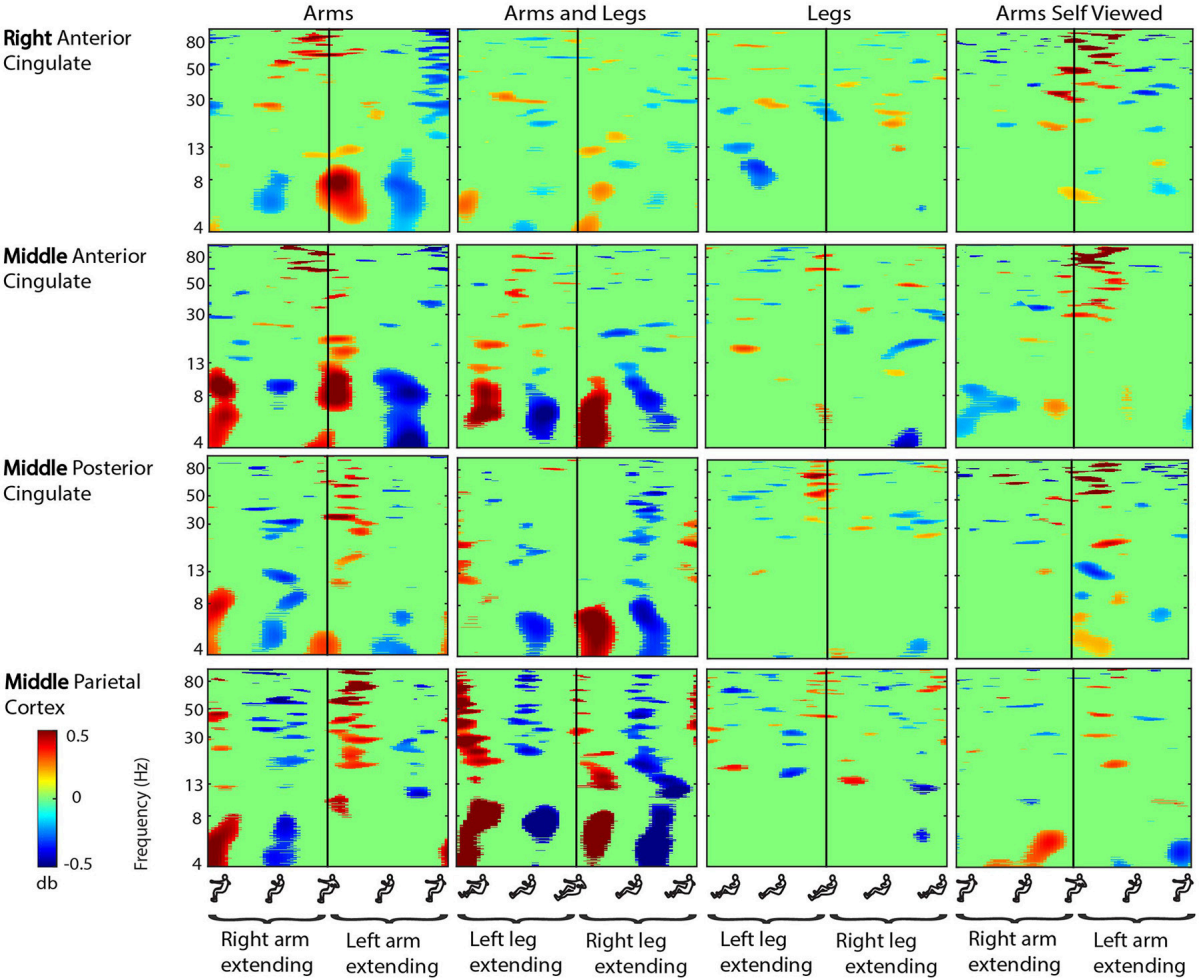
**Cingulate cortex ERSPs**. Event-related spectral perturbation (ERSP) plots showing change in spectral power during rhythmic upper and lower limb movement in the right anterior cingulate, the middle anterior cingulate, the middle posterior cingulate, and the middle parietal cortex. From left to right, subjects moved with their arms only, both their arms and legs, their legs only, and viewed video playback of themselves moving with just their arms. Each row represents a cortical area, and each column represents a condition. For all plots, red represents a power increase from baseline, and blue represents a power decrease from baseline. We set non-significant differences to 0 dB (green). All ERSPs start and end with the same limb fully extended. The figure outlines on the x axis indicate the phase of movement, and the written labels indicate when each limb was extending. Note: for the arms and legs condition, the left leg and right arm extended together, and vice versa.

There were seven clusters of electrocortical sources that had at least five subjects represented (Figure 2). Three clusters were located in the right, left, and middle premotor and supplementary motor area (Brodmann Area 6; Figure 3). There were also clusters in the right anterior cingulate (Brodmann area 32), the middle anterior cingulate (Brodmann Area 24), the middle posterior cingulate (Brodmann area 31), and the middle parietal lobe (Brodmann area 7; Figure 4). For a complete breakdown of functional areas and ICs, see Table 1.

**Table 1:** **Centroid location and IC breakdown for each cluster containing electrocortical sources from at least 5 of 10 subjects**.

**Functional area**	**Brodmann area of centroid**	**Subjects (#)**	**ICs (#)**
Right premotor and supplementary motor cortex	6	5	9
Middle premotor and supplementary motor cortex	6	9	22
Left premotor and supplementary motor cortex	6	7	17
Middle anterior cingulate	24	6	14
Right anterior cingulate	32	7	19
Middle posterior cingulate	31	8	17
Middle parietal cortex	7	7	11

In the premotor and supplementary motor cortex, spectral fluctuations occurred during the active conditions in the theta (4–8 Hz), alpha (8–13 Hz), beta (13–30 Hz), and gamma (30 Hz and above) bands (Figure 3). In the middle premotor and supplementary motor area, all active conditions elicited bilateral activity. Theta and low alpha band desynchronization occurred during approximately the middle 50% of the extension phase for either arm in the arms only condition, for either leg in the legs only condition, and for either arm-leg pair in the arms and legs condition. In the right and left premotor and supplementary motor cortex, these alpha and theta spectral power fluctuations showed some lateralization. Prominent desynchronization occurred in right premotor and supplementary motor cortex during approximately the middle 50% of the extension phase when the left leg was extending in both the arms and legs condition and the legs only condition. Prominent desynchronization occurred in left premotor and supplementary motor cortex during approximately the middle 50% of the extension phase when the left arm was extending in the arms only condition and when the right leg was extending in the legs only condition. In nearly all brain areas and frequency bands, when the Wilcoxon rank sum tests revealed significant differences in spectral power between active conditions, the legs condition had significantly less spectral power (Table 2). There were some similar spectral fluctuations during the self viewed arms condition, particularly in the middle premotor and supplementary motor cortex in the theta and low alpha band. However, overall, we did not detect robust or consistent fluctuations across similar pairs of conditions (i.e., arms vs. arms viewed). For the spectral fluctuation plots for all the viewed conditions, see Supplementary Figure 1.

**Table 2:** **Significant spectral differences between pairs of exercise conditions**.

	**Theta (3–8 Hz)**	**Alpha (8–13 Hz)**	**Beta (13–30 Hz)**	**Gamma (30–80 Hz)**
Right premotor and supplementary motor cortex		A > AL (*p* = 2.2e-6)	A > AL (*p* = 3.3e-4)	
		L > AL (*p* = 7.0e-4)	L > AL (*p* = 9.8e-5)	
Middle premotor and supplementary motor cortex	A > L (0.05)	A > L (*p* = 3.3e-7)	A > L (*p* = 0.007)	A > L (*p* = 0.01)
		A > AL (*p* = 2.1e-4)	A > AL (*p* = 0.02)	
		AL > L (*p* = 1.8e-4)		
Left premotor and supplementary motor cortex	AL > L (*p* = 0.05)			L > A (*p* = 0.03)
				AL > A (*p* = 1.6e-7)
				AL > L (*p* = 0.002)
Right anterior cingulate			A > AL (*p* = 0.001)	A > L (*p* = 0.004)
			L > AL (*p* = 0.04)	A > AL (*p* = 0.006)
Middle anterior cingulate	AL > A (*p* = 7.9e-7)	AL > A (*p* = 0.003)	AL > A (*p* = 1.1e-6)	A > L (*p* = 0.01)
	AL > L (*p* = 7.9e-7)	AL > L (*p* = 0.006)	AL > L (*p* = 9.4e-8)	AL > A (*p* = 6e-18)
				AL > L (*p* = 1.60e-26)
Middle posterior cingulate cortex	AL > A (*p* = 0.05)		A > L (*p* = 0.01)	A > L (*p* = 6.4e-19)
	AL > L (*p* = 8.1e-4)			AL > L (*p* = 5.0e-17)
Middle parietal cortex	A > L (*p* = 0.02)	A > L (*p* = 1.3e-4)	A > L (*p* = 0.01)	A > L (*p* = 7.3e-16)
		A > AL (*p* = 4.1e-6)	A > AL (*p* = 0.02)	A > AL (*p* = 0.006)
				AL > L (*p* = 3.0e-9)

**Figure 5:**
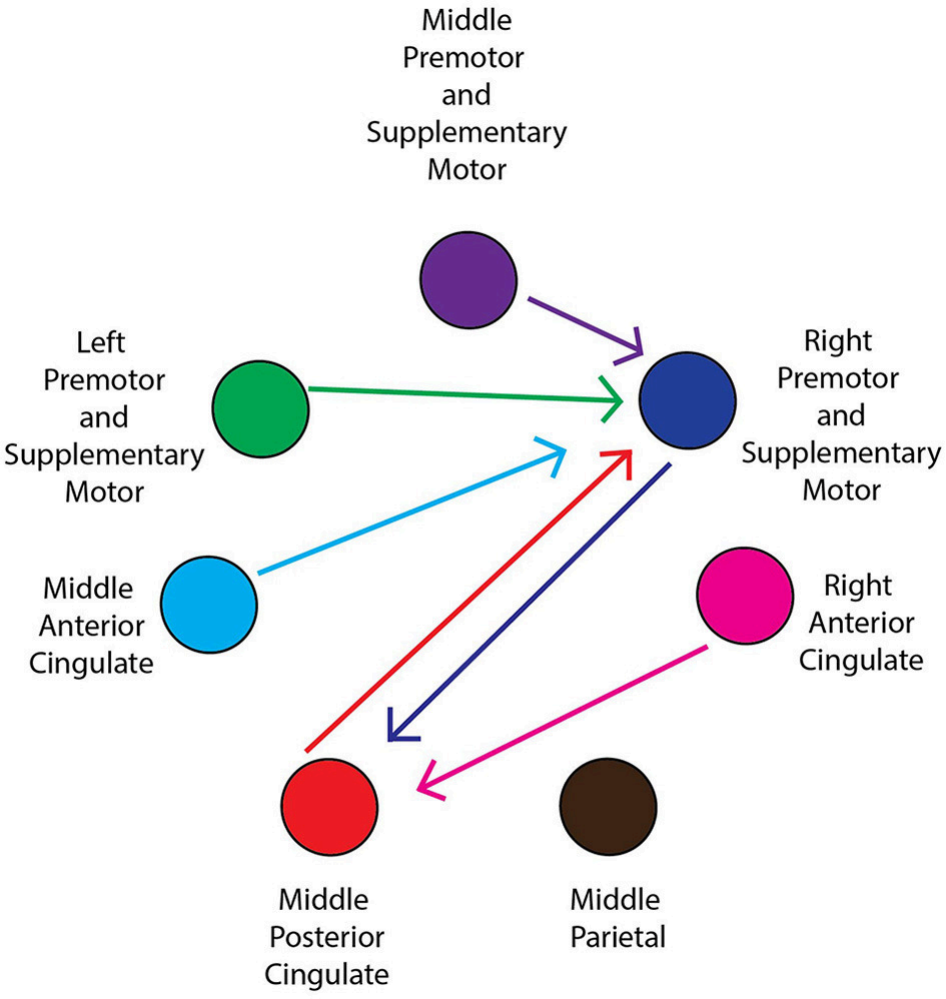
**General network connectivity**. Schematic of the cortical network active during active and viewed movement. Arrows between cortical areas indicate suprathreshold connectivities for at least half (5/10) of the conditions.

*Spectral comparisons for the three exercise conditions. A = Arms, L = Legs, and AL = Arms and Legs. Only pairs of conditions that had significantly different spectra for particular frequency bands (p < 0.05), as determined by Wilcoxon rank sum tests, are shown. Some spectral fluctuations also occurred during the viewed conditions in all brain areas, but they were generally smaller in magnitude or confined to the gamma band (Figure 5). Overall, these spectral fluctuations were not very similar for the exercise and viewed conditions*.

In the cingulate and parietal areas, spectral fluctuations occurred during active movement in the theta (4–8 Hz), alpha (8–13 Hz), beta (13–30 Hz), and gamma (30 Hz and above) bands (Figure 4). These fluctuations were much greater for both active arms conditions than for the active legs condition. In the majority of areas, theta and low alpha band desynchronization occurred during approximately the middle 50% of the extension phase when either arm was extending in the arms only condition and when either arm-leg pair was extending in the arms and legs condition. Theta and low alpha synchronization occurred at the transition points for the two active arms conditions. Again, we did not detect highly similar spectral fluctuations for the active arms and self viewed arms conditions. For the spectral fluctuation plots for all the viewed conditions, see Supplementary Figure 1. For spectral power comparisons between the active conditions broken down by frequency band, see Table 2.

### Connectivity results

For all conditions, pairs of cortical areas exhibited suprathreshold connectivity values (maximum connectivity values more than a standard deviation greater than the mean). Examining the connectivity patterns across all conditions (Table 3), a general picture of a movement-related neural network emerges. During every active and viewed movement condition, suprathreshold connectivity occurred between the middle anterior cingulate and the right premotor and supplementary motor cortex. For the majority of conditions, suprathreshold connectivity also occurred between several cortical areas (the middle premotor and supplementary motor cortex, the left premotor and supplementary motor cortex, and the middle posterior cingulate) and the right premotor and supplementary motor cortex, between the right premotor and supplementary motor cortex and the middle posterior cingulate, and between the right anterior cingulate and the middle posterior cingulate (Figure 5).

**Table 3:**
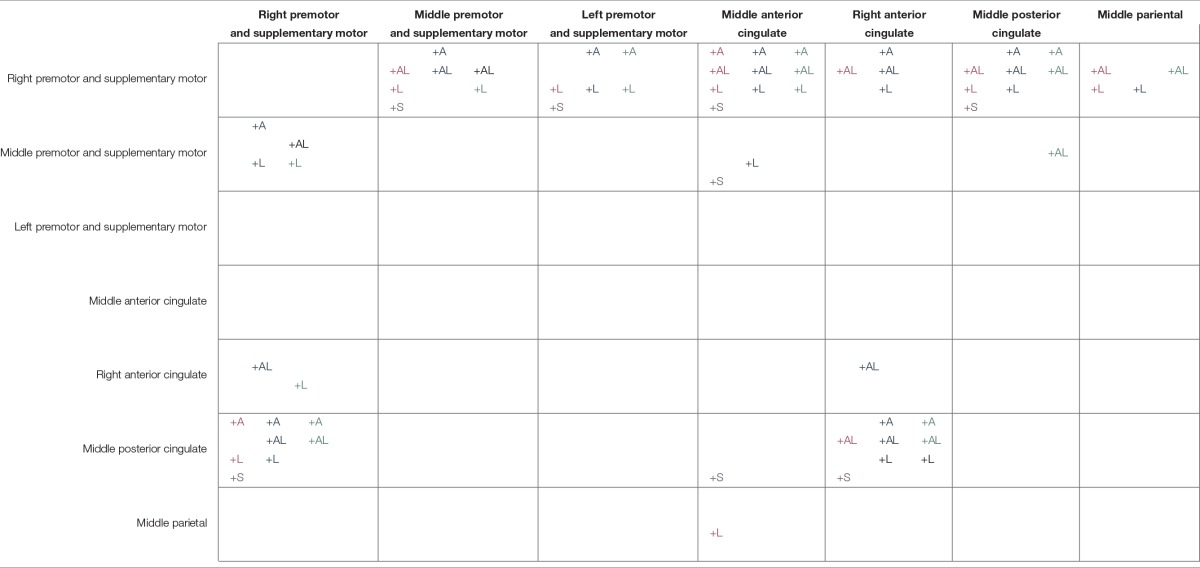
**Suprathreshold connectivity between pairs of cortical areas for the active movement the self-viewed movement, and the other-viewed movement conditions**.

+ = Supratheshold connectivity during active movement conditions.

+ = Suprathreshold connectivity during self-viewed movement conditions.

+ = Suprathreshold connectivity during other-viewed movement conditions.

+ = Suprathreshold connectivity during viewed stepper condition.

*A = Arms, AL = Arms and Legs, L = Legs, S = Stepper*.

There were also some differences between the conditions. The most suprathreshold connectivity values between pairs of cortical areas occurred during the self-viewed movement conditions (25 pairs). The least suprathreshold connectivity values between pairs of cortical areas occurred during the active movement conditions (15 pairs). The number of cortical pairs exhibiting suprathreshold connectivity was intermediate for the other-viewed movement conditions (18 pairs). See Table 3 for all cortical pairs exhibiting suprathreshold connectivity for each condition.

Connectivity grids showing the strength of directed transfer function connectivity for all frequencies and time points within a stride revealed that significant connectivity fluctuations occurred between many cortical areas for all active and viewed conditions (Figures 6–9). For connectivity grids for all the additional viewed conditions, see Supplementary Figures 2–7.

**Figure 6:**
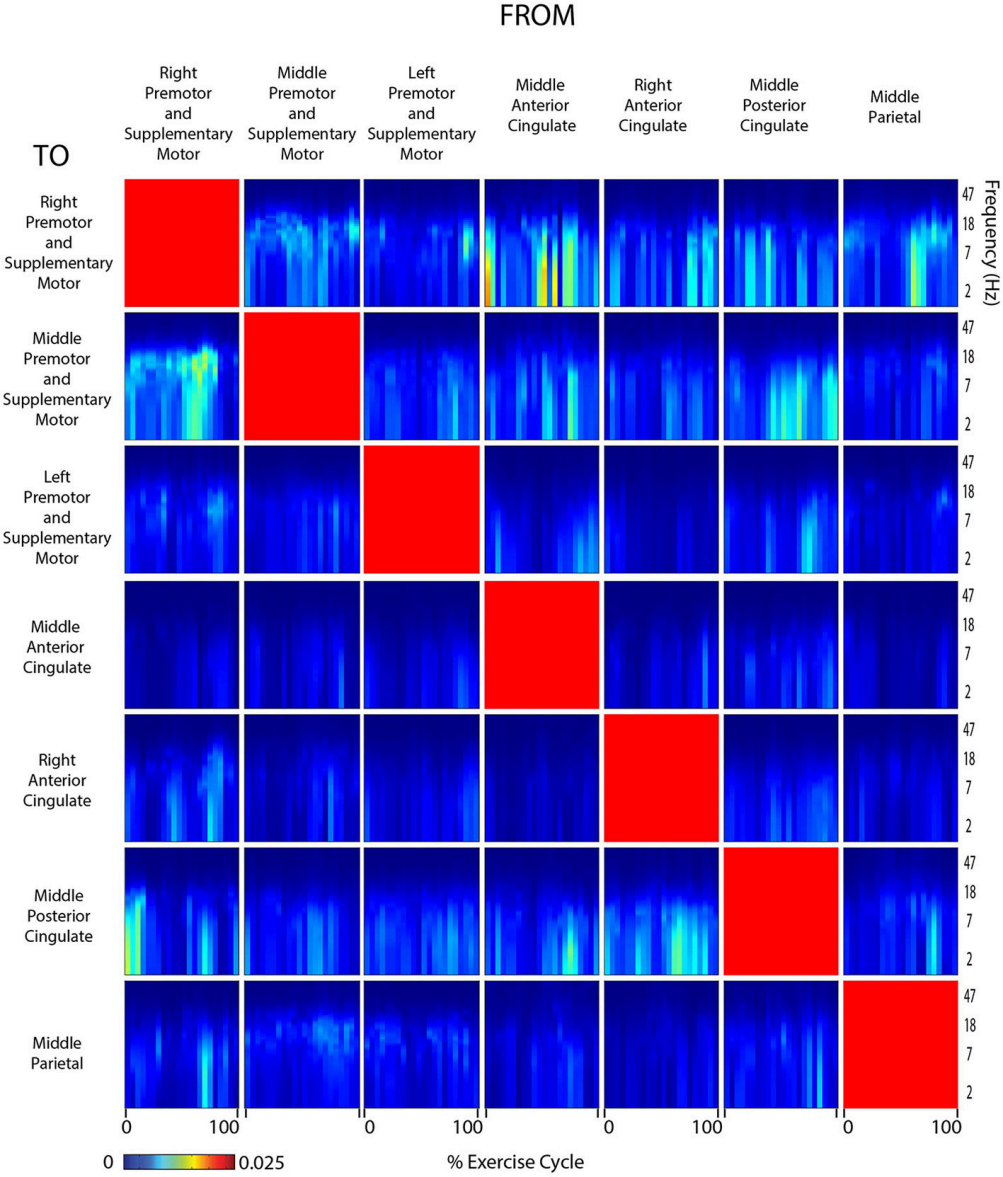
**Connectivity active arms**. Diagram showing directed transfer function connectivity values between pairs of cortical areas while the subject performed ACTIVE rhythmic movement using only the ARMS. Each individual plot starts and ends with the right arm fully extended. The numbers on the x-axis indicate the % of the movement cycle. We set non-significant differences to 0 (blue).

**Figure 7:**
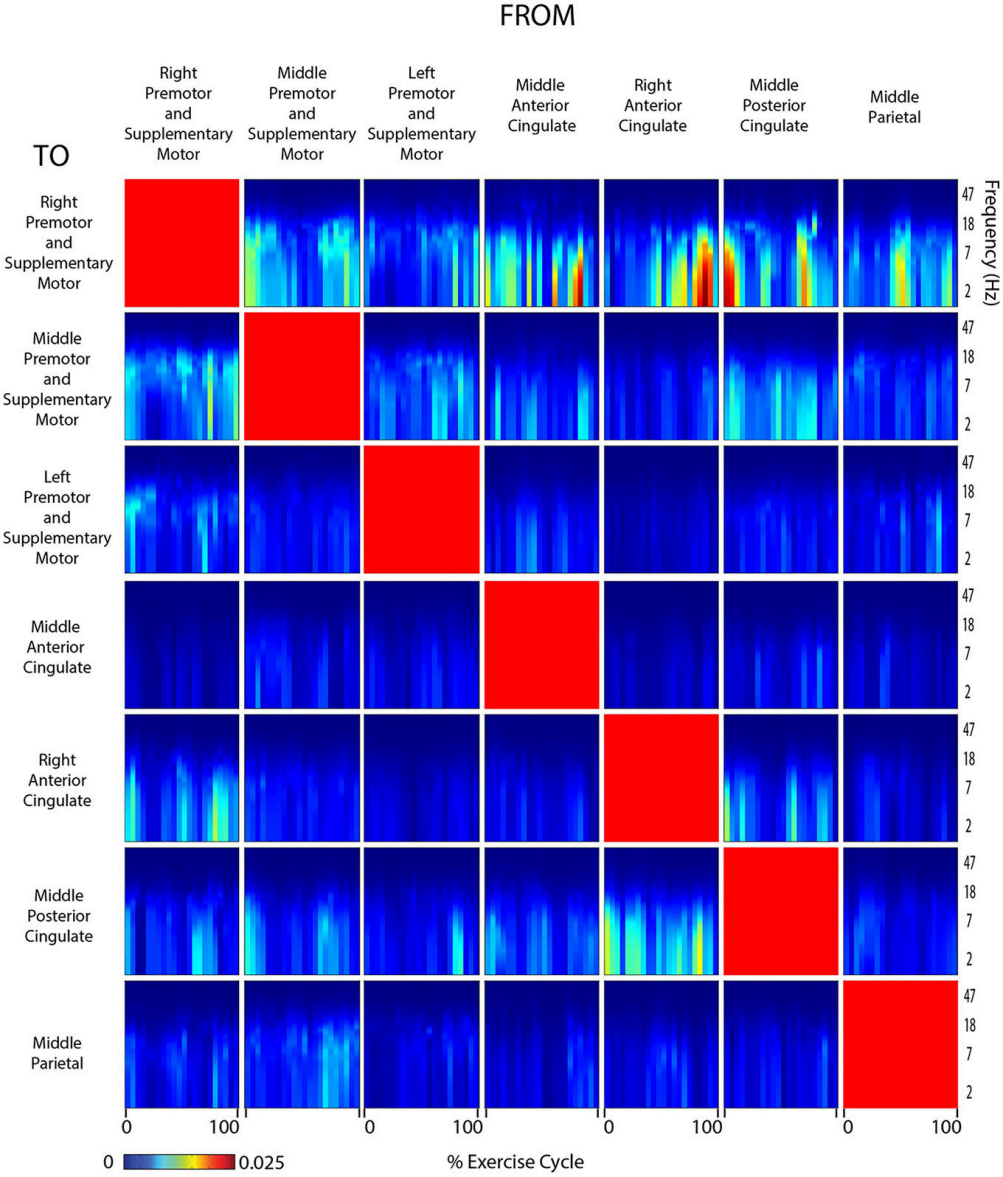
**Connectivity active arms and legs**. Diagram showing directed transfer function connectivity values between pairs of cortical areas while the subject performed ACTIVE rhythmic movement using the ARMS AND LEGS. Each individual plot starts and ends with the left leg fully extended. The numbers on the x-axis indicate the % of the movement cycle. We set non-significant differences to 0 (blue).

**Figure 8:**
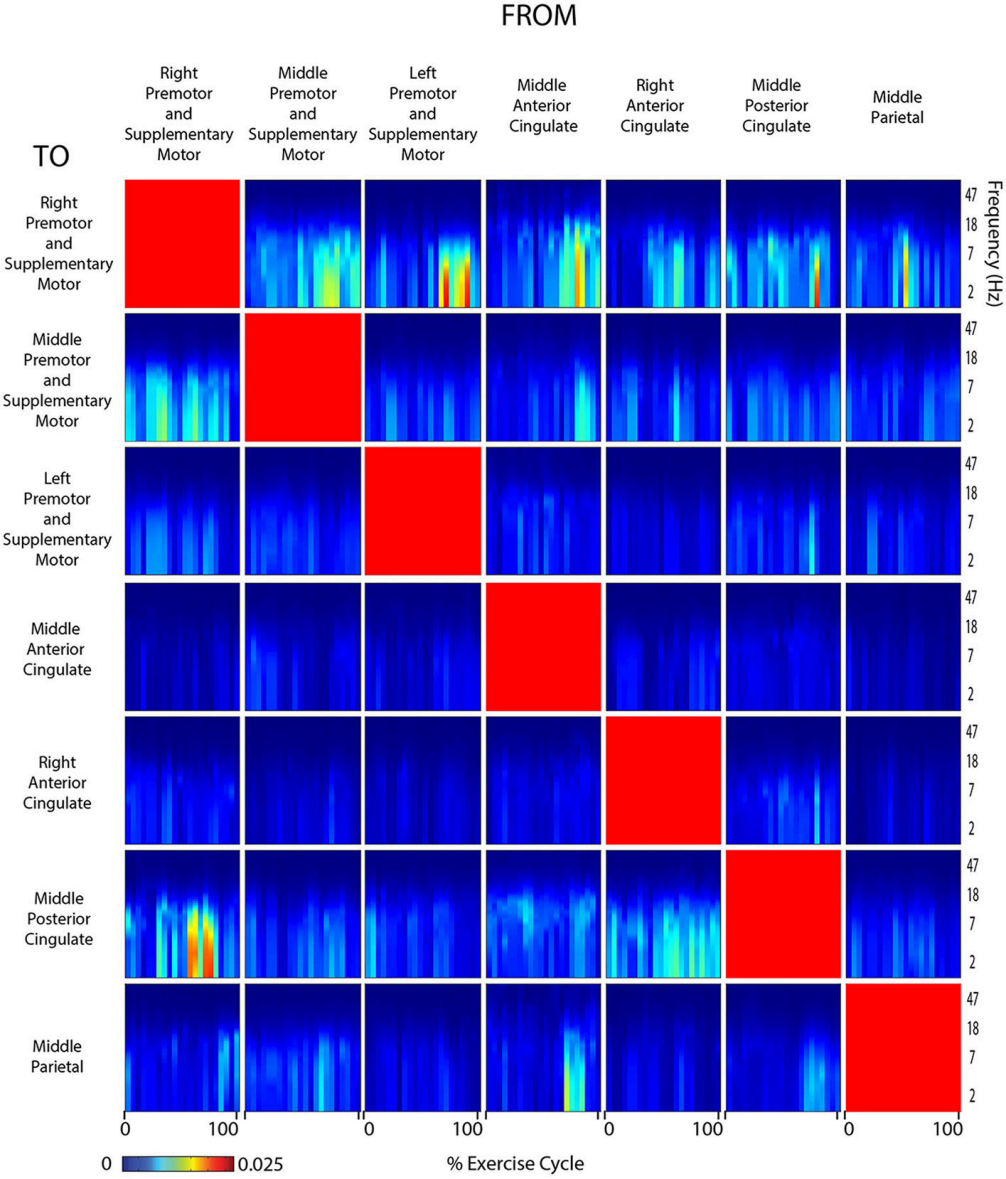
**Connectivity active legs**. Diagram showing directed transfer function connectivity values between pairs of cortical areas while the subject performed ACTIVE rhythmic movement using only the LEGS. Each individual plot starts and ends with the left leg fully extended. The numbers on the x-axis indicate the % of the movement cycle. We set non-significant differences to 0 (blue).

**Figure 9:**
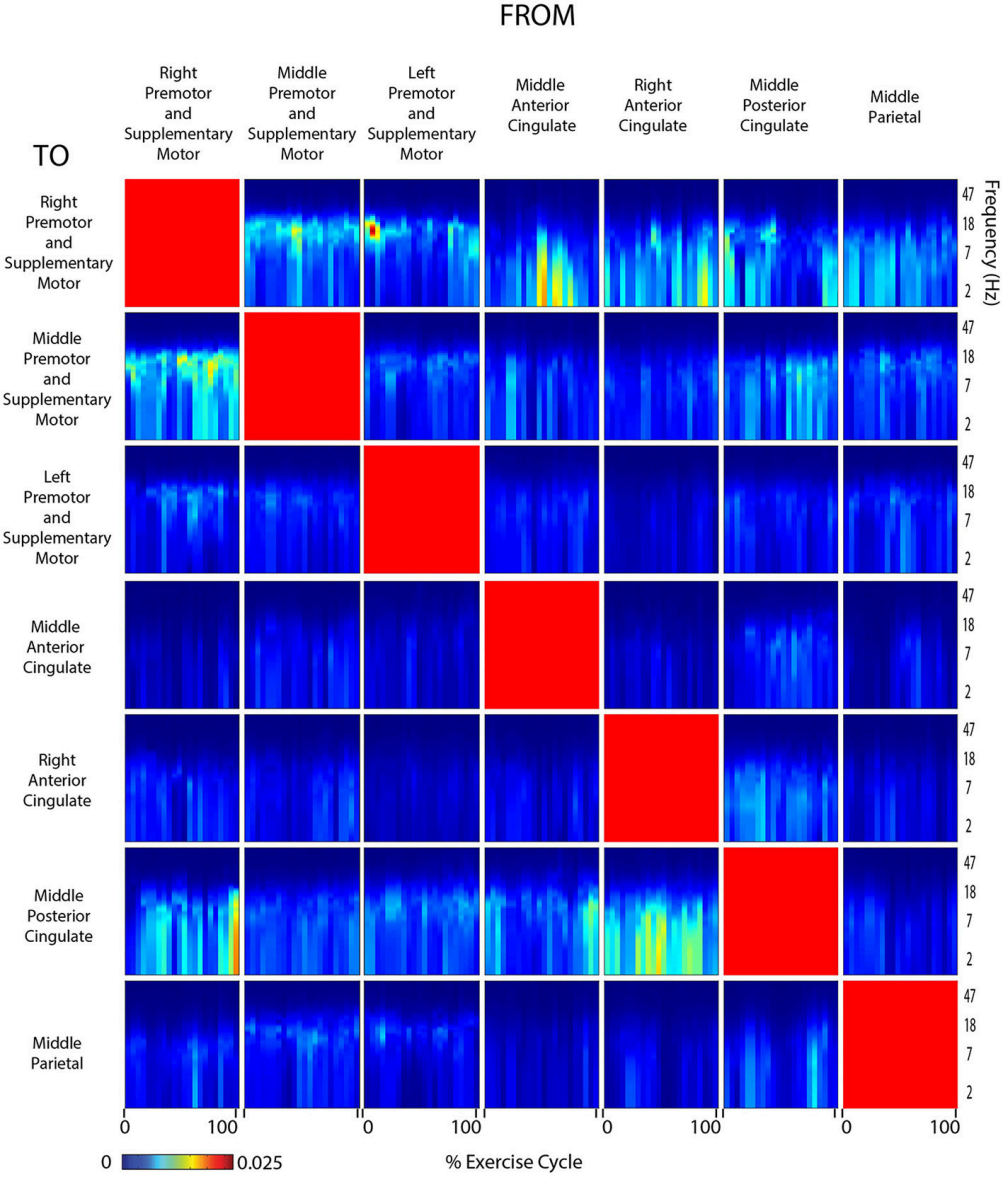
**Connectivity viewed arms self**. Diagram showing directed transfer function connectivity values between pairs of cortical areas while the subjects VIEWED a video of themselves exercising with only the ARMS. Each individual plot starts and ends with the viewed right arm fully extended. The numbers on the x-axis indicate the % of the movement cycle. We set non-significant differences to 0 (blue).

**Figure 10:**
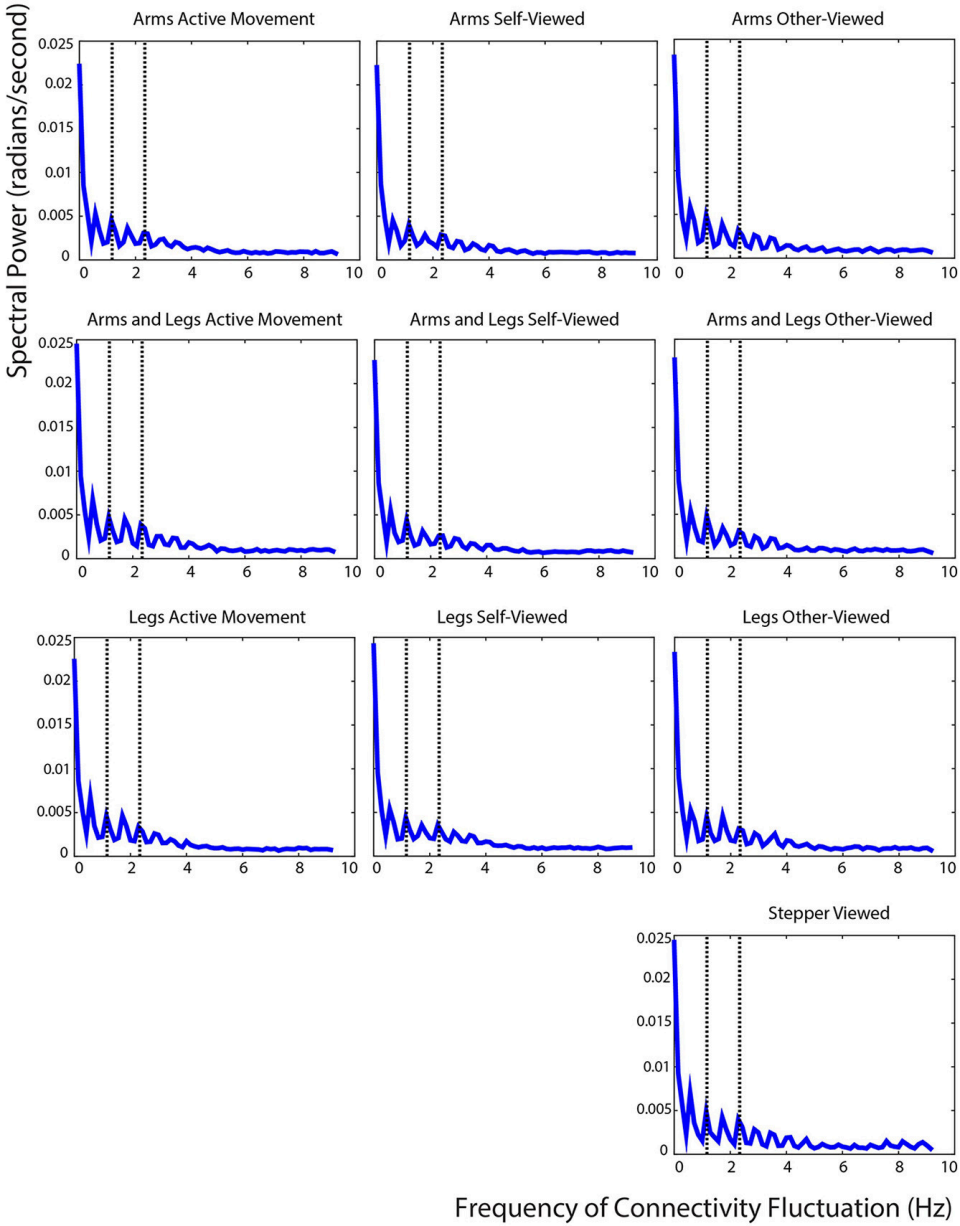
**Fast Fourier transform results**. Fast Fourier transform of the average connectivity over time across all IC pairs. The left column shows the values for the three active movement conditions, the middle column shows the values for the three self-viewed movement conditions, the right column shows the values for the four other-viewed movement conditions. The dotted lines show the subjects' stepping frequency and the second harmonic of that frequency.

The fast Fourier transform analysis showed that the connectivity strengths in our cortical network fluctuated at a predictable rate for both the active movement and the viewed movement conditions. Connectivity fluctuated rapidly, with the greatest peaks occurring below 3 Hz for all conditions. Peaks occurred at the harmonics of the movement frequency for both the active movement and the viewed movement conditions. For all conditions, a peak in connectivity fluctuation occurred at 0.5, 1, 1.5, and 2 times the movement frequency (1.17 Hz). Of particular interest is the fact that the peaks in connectivity fluctuation occurred at precisely the same frequencies for the active movement and the viewed movement conditions (Figure 10).

## Discussion

A widely distributed cortical network exhibits fluctuations in spectral power and effective connectivity during active and viewed rhythmic limb movements. This network includes the premotor and supplementary motor cortex, the anterior and posterior cingulate, and the parietal cortex. In the cingulate areas and the parietal cortex, the spectral fluctuations were significantly smaller or nearly absent during the legs only movement condition compared to either arm movement condition. This evidence suggests that rhythmic arm movements induce more cortical spectral activity than rhythmic leg movements. Consistent spectral fluctuations were not evident during the viewed conditions. However, effective connectivity analysis revealed that the strength and direction of information flow was similar between the active and viewed movement conditions. There was no strong difference in effective connectivity between the self viewed and other viewed movement conditions. For all the active and viewed conditions, the right premotor and supplementary motor cortex drove the network.

In the premotor and supplementary motor areas, spectral power fluctuations occurred most prominently during active rhythmic movements and less so during viewed movements. We found that the premotor and supplementary motor areas exhibited theta and alpha desynchronization when the contralateral limb was extending and synchronization when the limbs were switching directions. The presence of desynchronization may reflect increased cortical activation for movement production or sensorimotor processing (Pfurtscheller and Klimesch, 1991; Pfurtscheller et al., 1996; Pfurtscheller and Lopes Da Silva, 1999). The presence of synchronization at the transition points may reflect recruitment of the muscles needed to transition from flexion to extension (Jain et al., 2013). There were some similar spectral shifts during the viewed conditions. However, they were generally smaller, as in the middle premotor and supplementary motor area during the self viewed arm condition, but for the most part they were absent. This differs from previous EEG studies where spectral fluctuations were evident during viewed movement (Cochin et al., 1998, 1999; Calmels et al., 2006; Avanzini et al., 2012; Cevallos et al., 2015).

The spectral fluctuations seen during the active conditions in the motor cortex occurred primarily at lower frequencies than have been reported by previous studies of human walking (Gwin et al., 2011; Severens et al., 2012; Sipp et al., 2013; Nathan and Contreras-Vidal, 2016). During walking, there are many neural demands that are not present during a recumbent stepping task. The brain must coordinate foot placement, track the trajectories of all four limbs in space, and maintain balance. Comparatively, there is no balance component involved in recumbent stepping, and, for the most part, the limbs follow prescribed trajectories. The increased sensory, proprioceptive, and balance information processed by the brain during walking may account for much of the high frequency fluctuations seen in previous studies of human walking (Gwin et al., 2011; Severens et al., 2012; Sipp et al., 2013).

Spectral fluctuations also occurred during the active conditions in the cingulate areas and the parietal area. Similar to the premotor and supplementary motor areas, theta and alpha desynchronization occurred when the contralateral limb was extending, and synchronization occurred when the limbs were switching directions. Interestingly the spectral fluctuations in these areas were mainly apparent when the subjects moved their arms, and were much smaller or completely absent when the subjects moved just their legs. Past research has suggested that rhythmic leg movement is more tightly coupled to spinal neural centers than rhythmic arm movement. During arm and leg cycling in humans, leg cadence variability is less affected by changes in arm cycling cadence than vice versa (Sakamoto et al., 2014). An instantaneous change in arm cycling cadence has little effect on leg cycling cadence, but the converse is not true (Sakamoto et al., 2007). Our present findings support the notion that the legs are more tightly coupled than the arms to spinal neural networks and thus needs less descending input from areas such as the cingulate and parietal lobe, at least for a rhythmic, continuous task like recumbent stepping. The anterior cingulate area is primarily involved in error detection and correction (Bush et al., 2000; O'Connell et al., 2007; Walton et al., 2007). Past studies have found anterior cingulate spectral power fluctuations around foot placement during walking (Gwin et al., 2011), when there is a priority on monitoring potential errors that will affect gait stability. The legs exercise task had no active foot placement as the foot was always on the pedal, suggesting little need for cingulate error monitoring. Cyclic arm motion also had continuous effector-handle contact, but it is not similar to any normal locomotor task and thus could have been more closely monitored by the anterior cingulate. The middle parietal cortex is known to be involved in visuospatial processing (Harris et al., 2000). There are few occasions in everyday life when humans move the arms rhythmically without also moving the legs. Given the novelty of moving the arms rhythmically on their own, greater visuospatial processing may be required.

In our study, the right premotor and supplementary motor cortex was the central hub of information flow. It is well-established that the right hemisphere is the more spatially oriented of the two hemispheres (Joseph, 1988). The strongest evidence of this comes from patients who have suffered strokes on the right side of the brain. Hemineglect, or failure to perceive the contralesional side of the world, is typically more pronounced in patients with right hemisphere strokes than similar left hemisphere strokes (Bowen et al., 1999; Heilman et al., 2000). This suggests that the right hemisphere is centrally involved in constructing our perception of the space around us. Furthermore, the right hemisphere may control shifts in attention while viewing a scene. Studies with fMRI have reported right-lateralized ventral fronto-parietal activity during shifts in visual attention (Arrington et al., 2000; Corbetta et al., 2000). Seven of the ten conditions in this study involved a predominantly visual task with frequent shifts in attention from the left to right side of the viewed scene and vice versa. All of this may have accounted for the right hemisphere's prominent role in coordinating the communication of the interacting brain areas.

The results from our fast Fourier transform analysis of the connectivity data highlight similarities in the neural processing of the active movement and viewed movement conditions. The fluctuations in overall connectivity occurred at frequencies related to the movement. Specifically these fluctuations were prominent at 0.5, 1, 1.5, and 2 times the movement frequency. This observation strongly supports the relevance of connectivity analysis for providing insight into the true brain activity during the conditions. The prominent frequencies for greater connectivity fluctuations were similar for both active movement and viewed movement. There has been considerable recent debate about the possibility of motion artifact corrupting EEG signals during human movement (Castermans et al., 2014; Kline et al., 2015). There was very little head movement during the active stepping condition, but virtually no head movement during the viewed conditions. The fast Fourier transform analysis of the connectivity found very similar outcomes for all the conditions. The similarity between conditions indicates that the communication within the cortical network was related to the pace of the active or viewed movement. In addition, the most recent papers on head motion artifact in EEG during locomotion strongly suggest that standard processing methods remove the vast majority of motion artifact (Snyder et al., 2015; Nathan and Contreras-Vidal, 2016).

One of the limitations of our study was that subjects exercised at a self-reported challenging resistance level. As a result, we can only state that exercise with arms at a self-selected resistance elicits more cortical activity than exercise with legs at a self-selected resistance. Because we did not normalize absolute effort or force levels across participants and across conditions, we cannot report how these variables affected neural activity. Different amounts of resistance alter Blood Oxygenation Level Dependent (BOLD) signal in functional magnetic resonance imaging studies (Ward et al., 2008), but the same does not appear true with EEG spectral power. A previous study from our laboratory found no differences in event related spectral power fluctuations across a four-fold change in effort level for lower limb exercise (Gwin and Ferris, 2012). Two other studies using scalp EEG during exercise have also found either no differences in sensorimotor cortical activity over a four-fold range of effort (Dal Maso et al., 2012) or only a change in the gamma band of sensorimotor cortex activation over a four-fold range of effort (Fry et al., 2014). There are two published cycling studies that show EEG spectral power increases with cycling mechanical power output (Brummer et al., 2011; Schneider et al., 2013), but data from both of the studies actually show very little change with pedaling power. There is not a proportional change in EEG spectral power with cycling mechanical power in either study. Most of the measures show no change with cycling mechanical power. The authors increased cycling mechanical power across time in the experimental protocol, and late in the experiment did find at least one condition with high mechanical power output that had higher EEG spectral power than previous conditions. However, given that fatigue during cycling increases EEG spectral power (Enders et al., 2016), it seems more likely that the data from Brummer et al. and Schneider et al. can be explained best by fatigue rather than cycling mechanical power output. Our experimental paradigm randomized the order of conditions and used only comfortable levels of resistance that were not likely to promote fatigue.

There were several other limitations to our study. A second limitation was that we gave the subjects a fixation point during the viewed conditions, preventing them from freely scanning the scene as in past studies of motor observation. This gaze constraint may have limited the spectral fluctuations normally associated with viewed motion (Cochin et al., 1998, 1999; Calmels et al., 2006; Avanzini et al., 2012; Cevallos et al., 2015). Third, a visual cue was used to control pace in the active conditions, but there were no visual cues presented during the viewed condition. In the active conditions, cue following may have imposed additional cognitive load that was not present in the viewed condition, and this could have led to greater spectral activity in the active conditions. Fourth, in the viewed stepper condition, we identified supra-threshold connectivity that was similar to the three viewed motion conditions. We believe that the participants may have spontaneously engaged in motor imagery during this viewed condition. All participants had stepped on the recumbent stepper prior to viewing the control video of the recumbent stepper moving on its own. Given that motor imagery activates similar neural structures and pathways as motor execution (Filimon et al., 2007; Vogt et al., 2013), this could explain the unexpected result. Finally, contrary to our expectations, we did not find significant neural activity in the premotor cortex. There are many reasons why it may be absent. The spatial resolution of EEG is limited to a few centimeters. Furthermore, there is significant variation in neural anatomy between individuals, and in this study we used an average template brain to localize cortical sources rather than using personalized MRIs. It is possible that some of the cortical activity localized to the supplementary motor cortex could have come from the adjacent primary motor cortex. Future studies using subject-specific MRIs to provide finer resolution of motor cortical activity may find activity in the primary motor cortex.

Despite these limitations, the results of this study add new information to our understanding of human motor control and motor observation. This is among the first studies to report on spectral fluctuations in human cortex during actual and observed full-body motion. There is substantial interest in observed motion in the rehabilitation community. It has been suggested that action observation could improve motor rehabilitation in patients with chronic stroke, Parkinson's disease, or cerebral palsy (Buccino, 2014). In adults with upper-limb impairment following a stroke, significant improvements in functionality were seen after 18 consecutive days of action observation therapy (Ertelt et al., 2007). In children with cerebral palsy, a similar duration of action observation therapy increased spontaneous use of the affected hand (Buccino et al., 2012). Given the potential therapeutic benefits of viewed movement, knowledge of the differences in cortical connectivity during active and viewed movement could provide insight for rehabilitation after a brain injury. It might also suggest potential rehabilitation targets accessible through transcranial magnetic stimulation.

The results of our spectral analysis suggest that rhythmic arm movements are under greater descending cortical control, especially by the cingulate and parietal areas, than rhythmic leg movement. Furthermore, effective connectivity in a cortical network that is driven by the right premotor and supplementary motor cortex fluctuates at harmonics of the movement frequency during both active and viewed movement. These results suggest that a similarly interconnected neural network is in operation during both active and viewed movement. They illustrate that effective connectivity analysis can provide insight into brain network activity beyond what can be gained from traditional spectral analysis.

## Author contributions

JK, HH, KS, and DF designed the experiment. JK and HH carried out the experimental procedures. JK and KS performed the data analysis. JK, HH, KS, and DF wrote and edited the manuscript.

### Conflict of interest statement

The authors declare that the research was conducted in the absence of any commercial or financial relationships that could be construed as a potential conflict of interest.
