# Supplementary material for: Cortical Spectral Activity and Connectivity during Active and Viewed Arm and Leg Movement
Source: Front Neurosci. 2016 Mar 10;10:91. doi: 10.3389/fnins.2016.00091 (PMC4785182; doi:10.3389/fnins.2016.00091)
Supplement: Supplementary file 1 [file DataSheet1.DOCX]

**Supplementary figures**

**
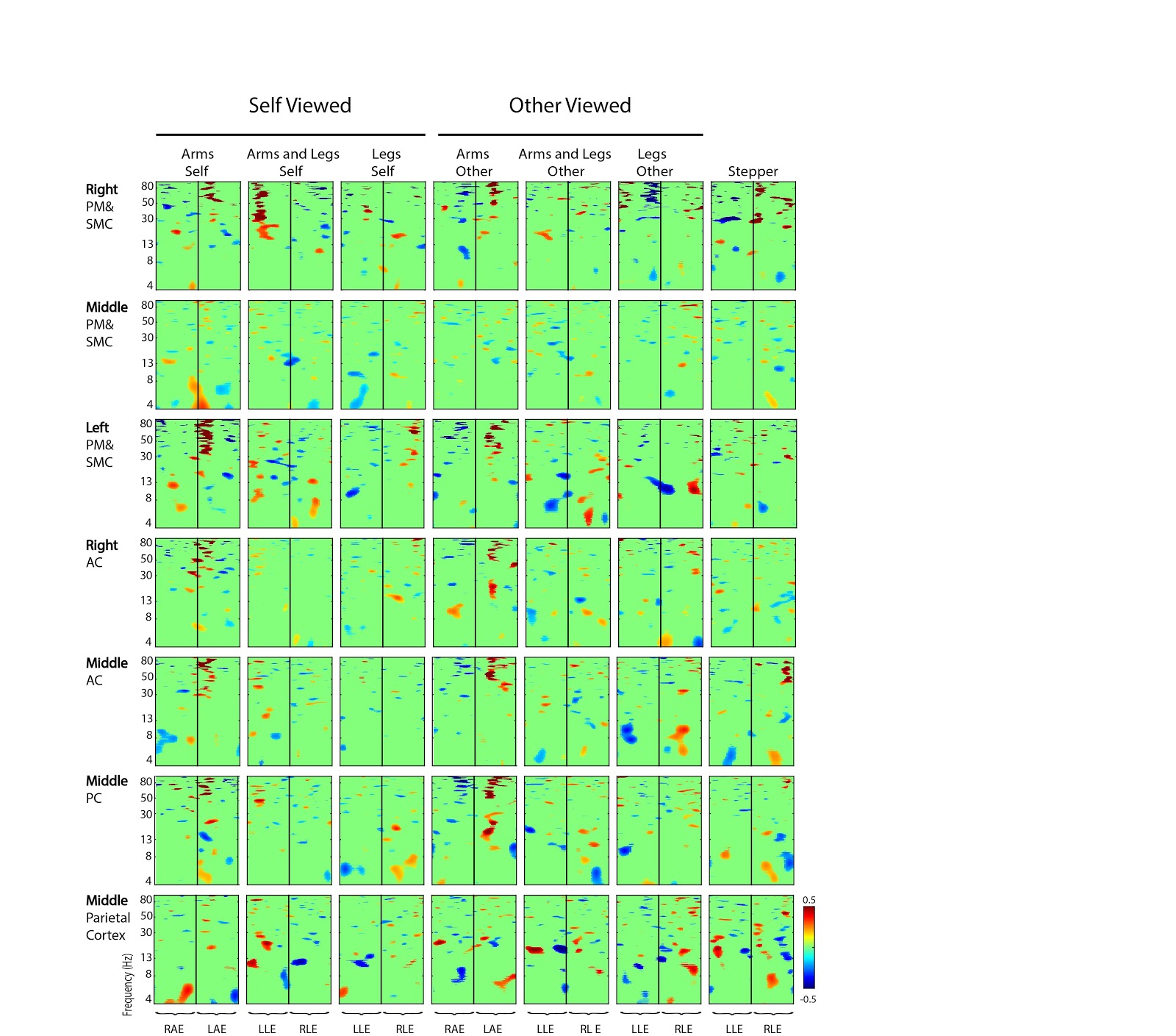
**

Supplementary Figure 1. All Viewed Movement ERSPs. Event-related spectral perturbation (ERSP) plots showing change in spectral power during viewed rhythmic arm and leg exercise in all electrocortical clusters. From left to right, in the first three columns subjects viewed themselves exercising with their arms only, with their arms and legs, and with their legs only. In the next three columns, they viewed another person exercising with her arms only, with her arms and legs, and with her legs only. In the last column (far right) subjects viewed the recumbent stepper moving on its own, with no one seated on it.  Each row represents a cortical area (PM&SMC=Premotor and Supplementary Motor Cortex, AC=Anterior Cingulate, and PC=Posterior Cingulate), and each column represents an exercise condition. For all plots, red represents a power increase from baseline, and blue represents a power decrease from baseline. We set non-significant differences to 0 dB (green). All ERSPs start and end with the same limb fully extended. The written labels indicate when each limb was extending. RAE=Right Arm Extending, LAE=Left Arm Extending, LLE=Left Leg Extending, RLE=Right Leg Extending. Note: for the arms and legs condition, the left leg and right arm extended together, and vice versa. For the stepper condition, the labels indicate the limb that would have been extending, had a person been seated in the stepper.

#
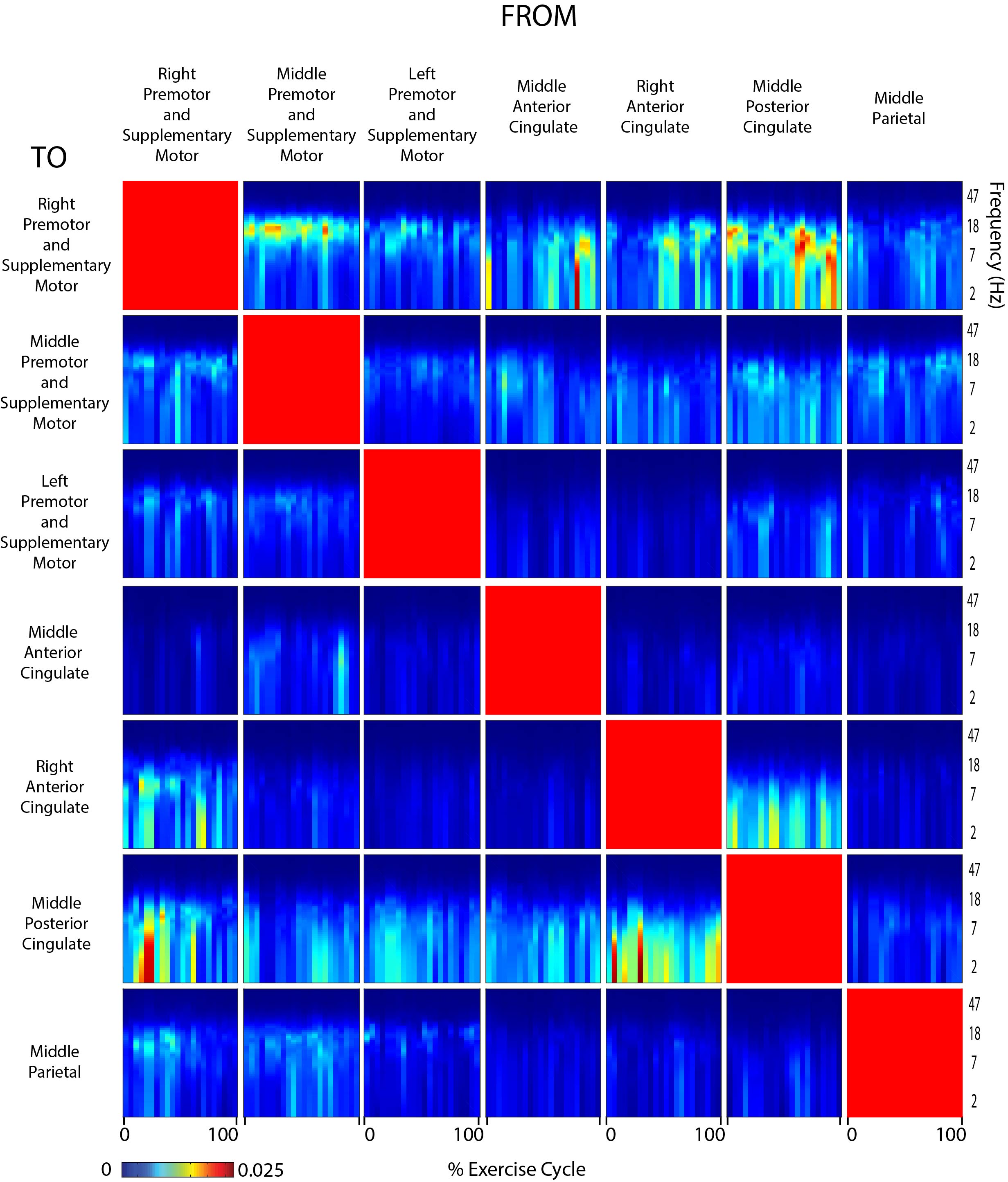


Supplementary Figure 2. Connectivity Self Viewed Arms and Legs.  Diagram showing directed transfer function connectivity values between pairs of cortical areas while the subjects VIEWED a video of THEMSELVES exercising with their ARMS and LEGS. Each individual plot starts and ends with the viewed left leg fully extended. The numbers on the x-axis indicate the % of the movement cycle. We set non-significant differences to 0 (blue).


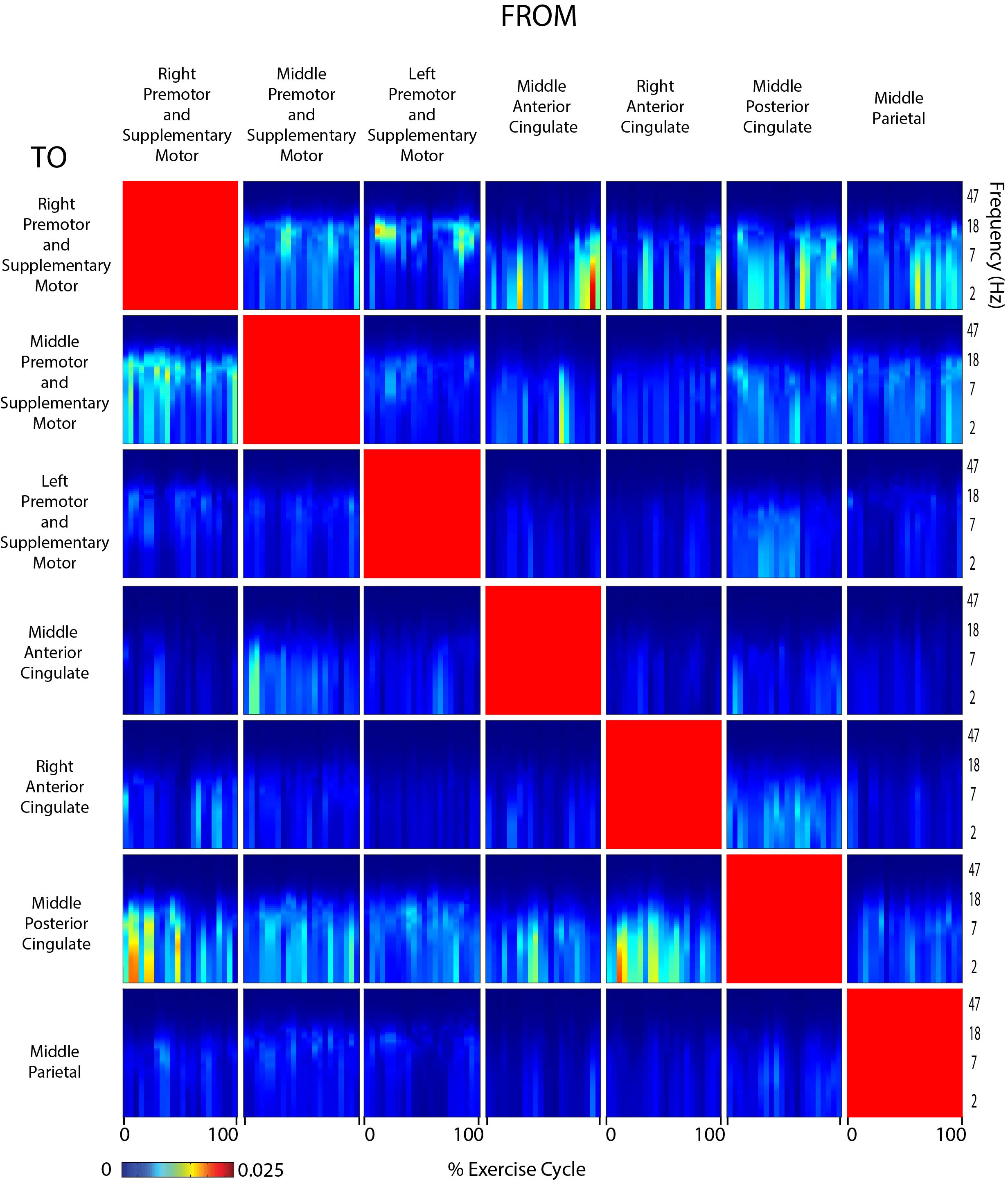


Supplementary Figure 3. Connectivity Self Viewed Legs.  Diagram showing directed transfer function connectivity values between pairs of cortical areas while the subjects VIEWED a video of  THEMSELVES exercising with only the LEGS. Each individual plot starts and ends with the viewed left leg fully extended. The numbers on the x-axis indicate the % of the movement cycle. We set non-significant differences to 0 (blue).

#
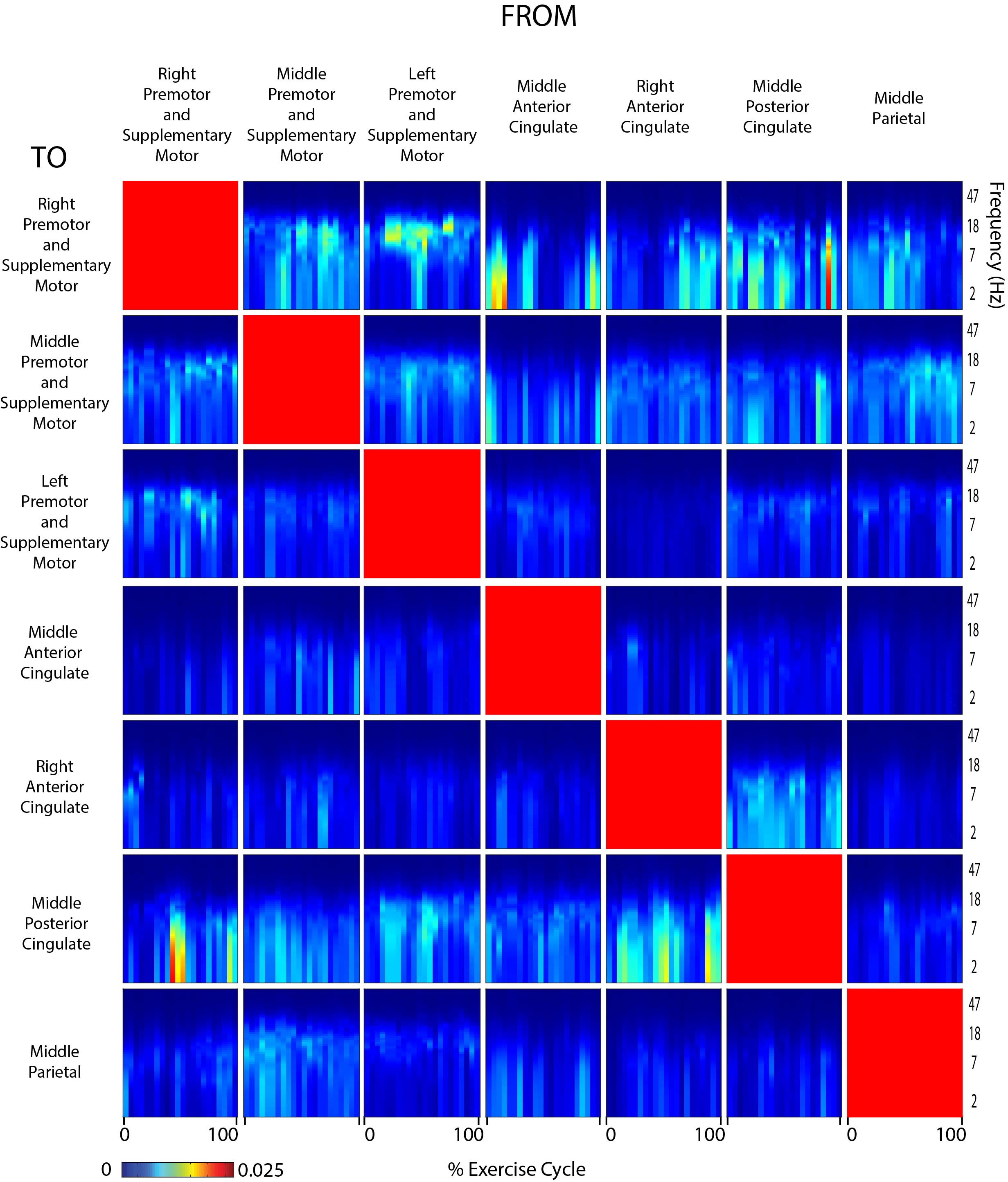


Supplementary Figure 4: Connectivity Other Viewed Arms. Diagram showing directed transfer function connectivity values between pairs of cortical areas for five second while the subjects VIEWED a video of ANOTHER PERSON exercising with only the ARMS. Each individual plot starts and ends with the viewed right arm fully extended. The numbers on the x-axis indicate the % of the movement cycle. We set non-significant differences to 0 (blue).


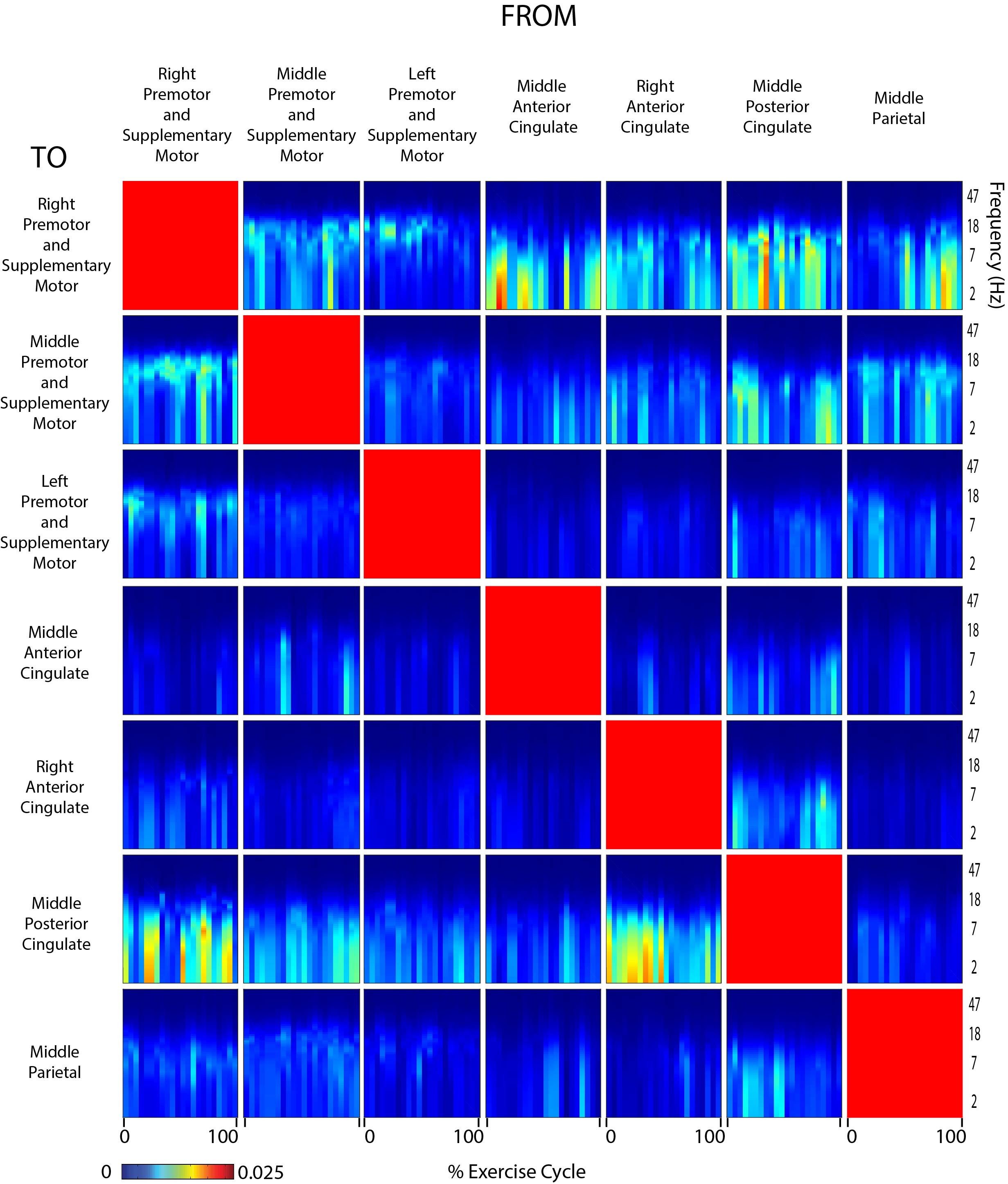


Supplementary Figure 5. Connectivity Other Viewed Arms and Legs.  Diagram showing directed transfer function connectivity values between pairs of cortical areas while the subjects VIEWED a video of ANOTHER PERSON exercising with their ARMS and LEGS. Each individual plot starts and ends with the viewed left leg fully extended. The numbers on the x-axis indicate the % of the movement cycle. We set non-significant differences to 0 (blue).


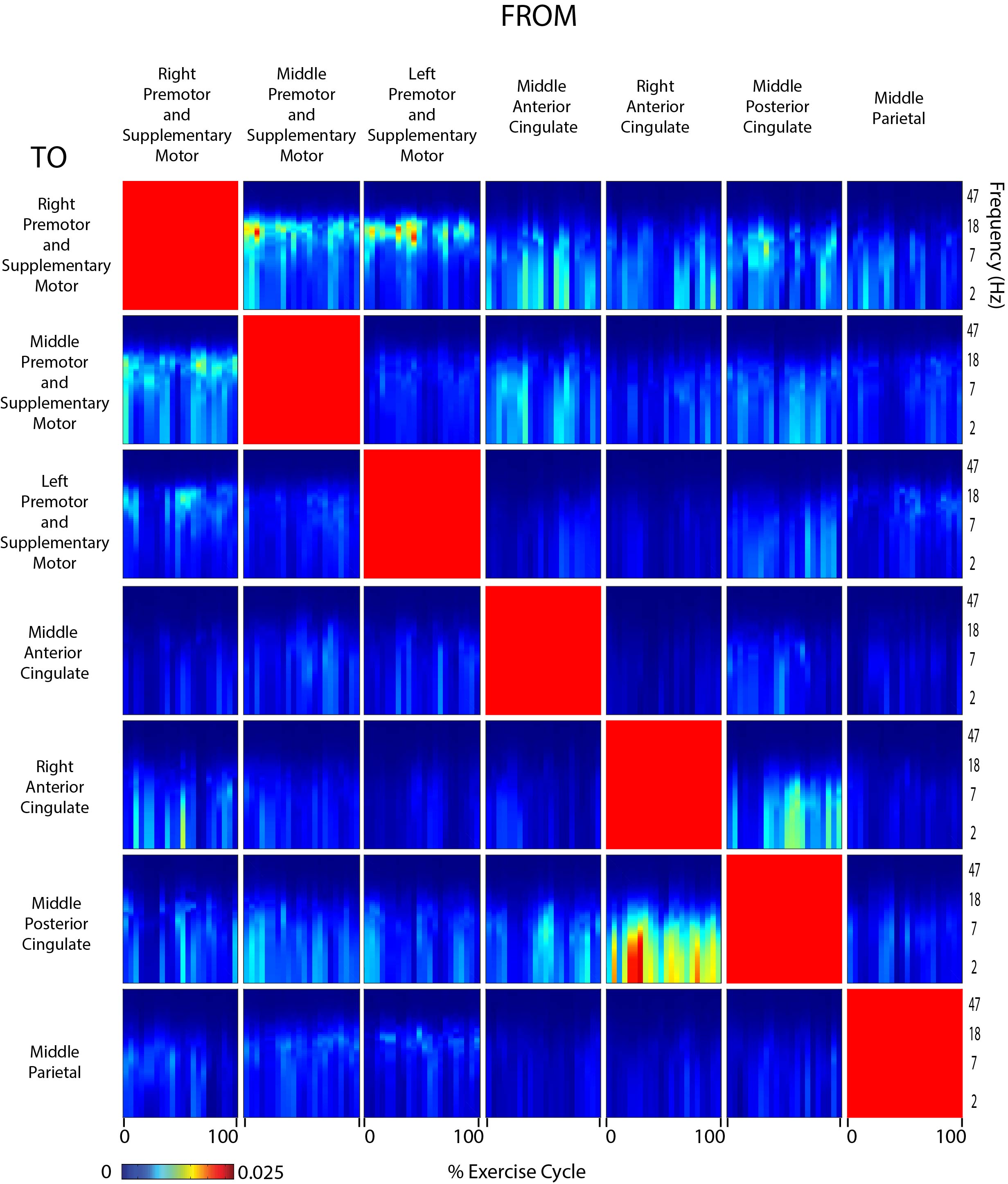


Supplementary Figure 6. Connectivity Other Viewed Legs. Diagram showing directed transfer function connectivity values between pairs of cortical areas while the subjects VIEWED a video of ANOTHER PERSON exercising with only the LEGS. Each individual plot starts and ends with the viewed left leg fully extended. The numbers on the x-axis indicate the % of the movement cycle. We set non-significant differences to 0 (blue).


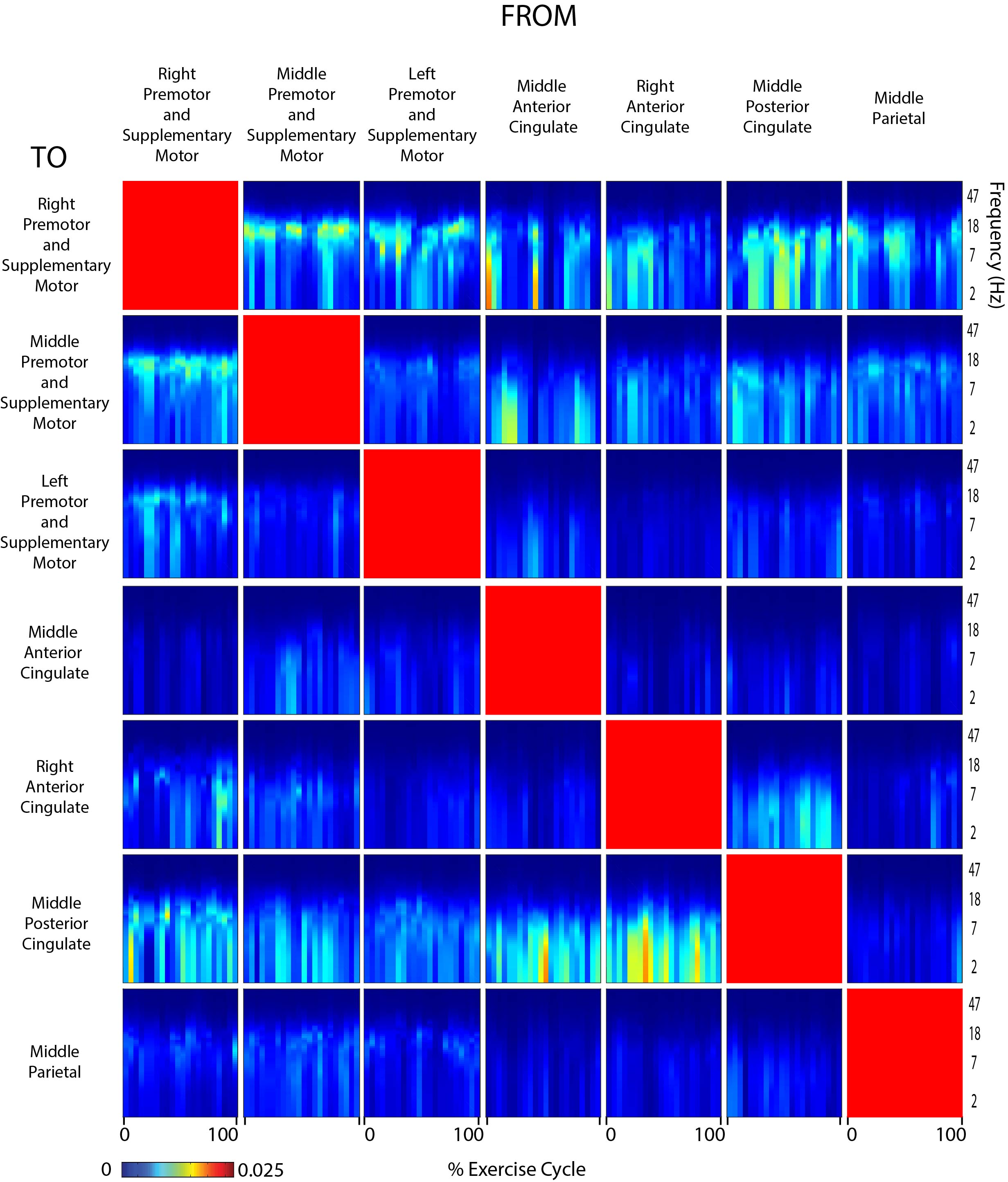


Supplementary Figure 7. Connectivity Viewed Stepper.  Diagram showing directed transfer function connectivity values between pairs of cortical areas while the subjects VIEWED a video of the RECUMBENT STEPPER moving on its own, with no one seated in it. Each individual plot starts and ends with where the left leg would have been fully extended, had a person been seated in it. We set non-significant differences to 0 (blue).
